# Low-density granulocytes display immature cells with enhanced NET formation in people living with HIV

**DOI:** 10.1038/s41598-023-40475-0

**Published:** 2023-08-16

**Authors:** Juwon Park, Logan S. Dean, Jack Heckl, Louie Mar Gangcuangco, Te-Kie Pedro, Michelle D. Tallquist, Todd B. Seto, Bruce Shiramizu, Dominic C. Chow, Cecilia M. Shikuma

**Affiliations:** 1 Hawaii Center for AIDS, John A. Burns School of Medicine, University of Hawai’i at Manoa, Honolulu, HI 96813 USA; 2 Department of Tropical Medicine, Medical Microbiology, and Pharmacology, John A. Burns School Medicine, University of Hawai’i at Manoa, Honolulu, HI 96813 USA; 3 Department of Cell and Molecular Biology, John A. Burns School Medicine, University of Hawai’i at Manoa, Honolulu, HI 96813 USA; 4 Center for Cardiovascular Research, John A. Burns School of Medicine, University of Hawai’i at Mānoa, Honolulu, HI 96813 USA; 5 The Queen’s Medical Center, Honolulu, HI 96813 USA; 6 Department of Medicine, John A. Burns School of Medicine, University of Hawai’i at Manoa, Honolulu, HI 96813 USA

**Keywords:** Infectious diseases, Inflammation, Innate immune cells, Translational immunology, HIV infections, Translational research, Immunopathogenesis, Inflammation

## Abstract

While the protective role of neutrophil extracellular traps (NETs) in limiting human immunodeficiency virus (HIV) spread to susceptible cells has been documented, there is comparatively little insight into whether NET formation is harmful in people living with HIV (PLWH). To gain insight into neutrophil dysregulation and the pathological role of NETs in HIV, we examined expressions of NET-associated markers [cell-free DNA (cfDNA) and citrullinated histone H3 (CitH3)] in the plasmas from a cohort of the Hawaii Aging with HIV-cardiovascular and HIV-seronegative (HIV−) individuals. In a subset of participants, circulating low-density granulocyte (LDG) levels and their maturation and activation status were analyzed via flow cytometry. We demonstrated higher plasma levels of CitH3 in PLWH compared to HIV- individuals. LDGs from PLWH had heightened CD66b, but reduced CD16 expression. The percentages and counts of CD10^+^ LDGs were significantly decreased in PLWH. In addition, the CD16^Lo^ LDG subsets were enriched in PLWH, compared to HIV− group, indicating that immature LDGs are increased in PLWH. Moreover, LDGs from PLWH exhibited significantly higher NET forming capacity. In summary, our study presents evidence that LDGs from PLWH on ART display an immature and altered phenotype with increased NET formation. Among PLWH, plasma NET levels as well as LDG parameters correlated with blood markers for inflammation and coagulation, suggesting that neutrophil activation and NETs may exert proinflammatory and coagulation effects. Our data provide insights into the pathologic role of LDGs at least in part mediated through NET formation in PLWH.

## Introduction

Human immunodeficiency virus (HIV) infection is a major public health burden, affecting approximately 38.4 million people globally with 1.5 million newly infected individuals in 2021^1^. With the advent and combination of antiretroviral therapy (ART), HIV infection can be better managed, substantially improving the life expectancy of people living with HIV (PLWH)^2,3^. Although substantial progress in preventing the progression of HIV infection to acquired immunodeficiency syndrome (AIDS) has been made, PLWH on suppressive ART experience morbidities associated with diabetes^4^, malignancies^5^, and cardiovascular disease (CVD)^6,7^. Age and duration of HIV infection have been shown to be associated with the incidence of non-AIDS comorbidities (NACMs)^8–11^. As the population of older PLWH (age ≥ 50 years) is anticipated to rise, the proportion of NACMs and NACMs-related deaths is also projected to increase^12^. Thus, research is needed to decipher the biological mechanisms linking HIV infection, even when well controlled, and NACMs, leading to the identification of potential therapeutic targets to reduce the global burden of NACMs.

While traditional non-AIDS risk factors and ART toxicities are associated with NACMs^11,13^, emerging evidence postulates that HIV-associated inflammation and persistent immune activation are important contributing factors for disease development^14–18^. Neutrophils, the most abundant leukocytes, are considered to be the front-line defender during inflammation and infection. Neutrophils respond to invading microorganisms and destroy them through their antimicrobial mechanisms, such as degranulation, phagocytosis, and the release of nuclear DNA in the form of neutrophil extracellular traps (NETs)^19,20^. Neutrophil’s protective role during viral infection is being increasingly recognized^21–23^. Regarding HIV infection, studies have demonstrated that NETs inhibited HIV production in macrophages^24^ and genital neutrophil-derived NETs play pivotal roles in inactivating HIV and preventing subsequent infection of CD4^+^ T cells^21^. In addition to their classic role in antimicrobial control, neutrophils also exert immunoregulatory functions and display phenotypic and functional plasticity^25,26^. Neutrophil dysfunction has been associated with clinical deterioration in various diseases^27–29^. Neutrophils isolated from PLWH with an increased inflammatory status were “hyperactivated” compared to PLWH with no inflammatory status^18^, demonstrating that the chronic activation state of the immune system in PLWH on stable ART supports neutrophil activation^16,18^. Furthermore, evidence has emerged that NETs are essential drivers in the pathophysiology of inflammatory and chronic diseases, including CVD^27–29^.

In general, expansion of immature neutrophils in the peripheral blood is indicative of infection^30,31^. However, increased immature form of neutrophils in the circulation has been reported in cancers^32^ and inflammatory diseases including sepsis and rheumatoid arthritis (RA)^33,34^. Multiple studies have indicated that immature neutrophils exhibit impaired efficacy of microbe clearance and altered immunoregulatory function, thereby contributing to disease progression and poor prognosis^32,35^. Low-density granulocytes (LDGs) are a subpopulation of neutrophils that co-exist within peripheral blood mononuclear cells (PBMCs) that can be isolated by density gradient separation^36^. LDGs are composed of immature and mature neutrophils in patients with chronic diseases^36,37^. LDGs have been shown to have an enhanced ability to form NETs^38^ and elevated LDG levels contribute to the pathogenesis of diseases involving various aspects of pathophysiology, including inflammation, vascular permeability, and thrombosis^29,39,40^. HIV-infected treatment naïve individuals have significantly higher myeloid-derived suppressor cell (MDSCs) levels, a considerable part of LDGs’ functionality compared to healthy controls^41^. Furthermore, CD66b, CD63, CD11b, and arginase 1 expression were increased on LDGs from treatment naïve individuals, suggesting an activated/degranulation phenotype^42^. To better understand neutrophils’ detrimental role in HIV, we analyzed plasma levels of NET formation in this study. We also characterized circulating LDG levels and their phenotype in PLWH on ART, comparing them to HIV-seronegative (HIV−) individuals. We additionally assessed the relationship between LDG parameters, markers for NETs, inflammation, and coagulation.

## Results

### Study participant description

We utilized plasma samples collected from PLWH who had available data on inflammatory biomarkers related to CVD as shown in Table 1. A total 141 subjects, (PLWH; n = 88 and HIV−; n = 53) were included. PLWH participants were slightly younger than HIV-seronegative (median age of 49 vs 55 years, p = 0.03). Gender, ethnicity, body mass index (BMI), and history of co-morbidities did not differ between the two groups (Table 1). Most participants (89.0%) had undetectable HIV RNA and the median CD4^+^ T cell count was 469 (294.5–605.5) cells/mm^3^. HIV infection is associated with profound effects on hematopoiesis and hematological abnormalities, and these abnormalities are commonly observed in PLWH. Although PLWH (20.5%) had observed neutropenia (absolute neutrophil count less than 2000/μL) and they were more likely to have reduced white blood cell (WBC) numbers, no significant differences in leukocyte, red blood cell (RBC), platelet, and neutrophil counts were found between HIV− and PLWH.

**Table 1:** Clinical and laboratory characteristics of PLWH and HIV-seronegative individuals.

	HIV− seronegative (N = 53)	PLWH (N = 88)	P-value
Demographics			
Age	55.0 [47.5–60.0]	49 [45–47]	0.03*
Male	46 (86.8%)	77 (87.5%)	0.90
Caucasian	34 (64.2%)	52 (59.1%)	0.55
Co-morbidities			
Body mass index (kg/m^2^)	26.7 [22.9–29.8]	25.8 [23.9–27.9]	0.51
Past cigarette smoking	28 (54.9%)	54 (61.4%)	0.45
Current cigarette smoking	8 (15.1%)	16 (18.2%)	0.64
Diabetes	2 (3.8%)	8 (9.1%)	0.32
Hypertension	13 (24.5%)	30 (34.1%)	0.23
High cholesterol	19 (35.8%)	38 (43.2%)	0.39
History of myocardial infarction	1 (1.9%)	4 (4.5%)	0.65
History of stroke	0 (0%)	2 (2.3%)	0.53
Laboratory parameters and NET formation markers			
CD4^+^ T cell count (cells/μL)	–	469 [294.5–605.5]	–
Hemoglobin (g/dL)	14.2 [13.6–15.0]	14.5 [13.5–15.45]	0.44
Platelet count (× 10^9^/L)	216 [180–256.5]	209 [175–233]	0.06
Total leukocyte count (× 10^9^/L)	5.20 [4.32–5.98]	5.1 [4.2–6.03]	0.59
Absolute neutrophil count (× 10^3^/μL)	3.02 [2.19–3.61]	2.74 [2.35–3.73]	0.82
CitH3 (ng/mL)	2.68 [1.96–5.28]	9.85 [4.64–13.0]	< 0.001*
cfDNA (μg/mL)	0.71 [0.67–0.74]	0.68 [0.56–0.77]	0.06

Values presented are median [quartile 1, quartile 3] and n (%), as appropriate.

*Statistically significant; P-values calculated using Chi-square test or Fisher’s exact test for proportions and Mann Whitney U test for medians.

### Circulating NET levels in PLWH

**Figure 1:**
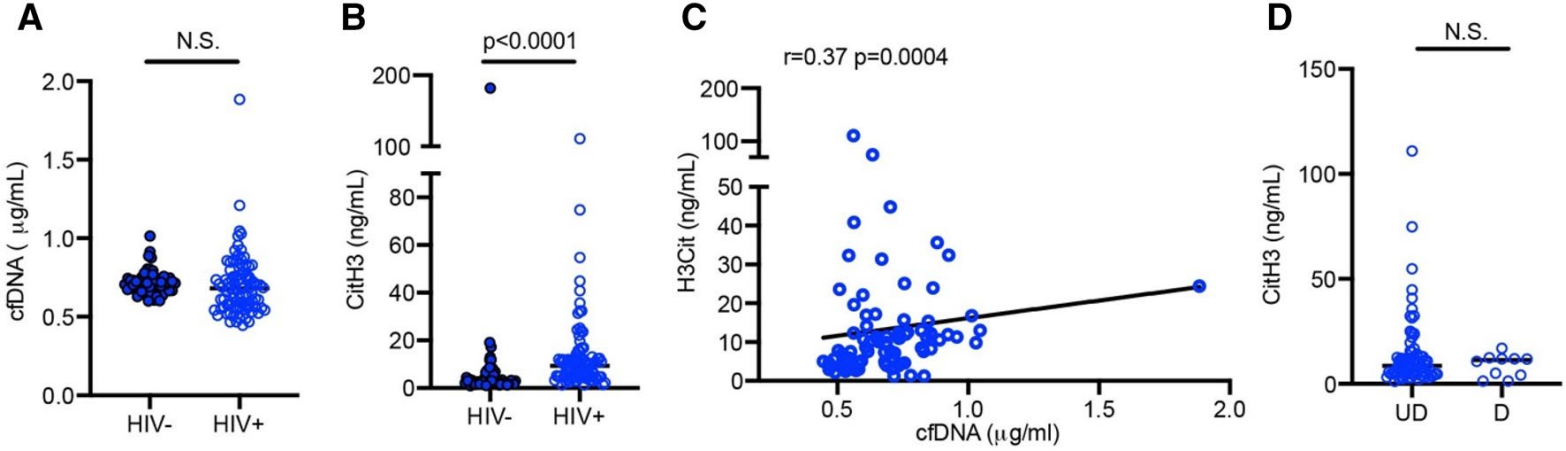
PLWH had higher levels of NET-associated markers in plasma compared to HIV− individuals. (**A,B**) Plasma samples from PLWH, referred hereafter as HIV+ (n = 88) and HIV− individuals (n = 58) were assessed for cfDNA (**A**) and CitH3 (**B**). (**C**) Correlation between CitH3 and cfDNA in PLWH. (**D**) Plasma levels of CitH3 in PLWH with/without detectable viremia. *UD* undetectable, *D* detectable. Spearman’s rank correlation was calculated. (**A,B,D**) Mann–Whitney U test, *N.S.* non-significant.

To measure NET-associated markers in PLWH (referred hereafter as HIV+ in figure) and compare them with HIV− individuals, cfDNA and CitH3 were quantified in the plasma. There were no differences in plasma cfDNA levels between HIV− and PLWH (0.71 vs 0.68 µg/mL) (Fig. 1A). However, PLWH had over a threefold increase in the median level of CitH3 compared to HIV- individuals (9.85 ng/mL vs 2.68 ng/mL, p < 0.0001) (Table 2 and Fig. 1B). In PLWH, there was a positive correlation between cfDNA and CitH3 (r = 0.37, p = 0.0004) (Fig. 1C). To evaluate if PLWH with detectable viral loads have increased circulating NETs, we compared CitH3 levels in PLWH with/without detectable viremia. Plasma levels of CitH3 were not significantly different regardless of detectable viral loads (Fig. 1D). PLWH often have observed hematological abnormalities, even in individuals under highly active ART^43^, affecting both red and white blood cells, as well as platelets. Therefore, we compared peripheral blood leukocyte counts between groups and determined if blood cell abnormalities in PLWH affects the quantity of NET markers in circulation. There were no differences in hematological parameters, including red blood cell (RBC), total leukocyte, absolute neutrophil, and platelet counts (Table 1). When we analyzed associations of circulating NET markers with hematological variables in PLWH, cfDNA was weakly correlated with total leukocyte and absolute neutrophil counts (r = 0.298, p < 0.01 and r = 0.278, p < 0.01, respectively). However, we did not observe associations between CitH3 and total leukocytes, absolute neutrophils, or platelet counts (Table 2). Evidence over the last decade suggests that NETs are a primary contributor to CVD development. We attempted to further investigate if circulating NETs are associated with inflammatory markers related to CVD. Notably, plasma CitH3 levels were positively correlated with cytokines, such as C-reactive protein (CRP), E-selectin, Interleukin (IL)-6, IL-1β, IL-10, and myeloperoxidase (MPO), as well as markers associated with coagulation; fibrinogen, serum amyloid A (SAA), and plasminogen activator inhibitor-1 (PAI-1) (Table 2). This data indicate that NETs likely contribute to inflammation in PLWH.

**Table 2:** Spearman’s correlations between NET markers and hematological parameters and soluble biomarkers in PLWH.

	CitH3 (r value)	cfDNA (r value)
Hematological parameters		
Total leukocyte count	0.095	0.298**
Absolute neutrophil count	0.031	0.278**
Platelet count	0.129	0.061
Soluble biomarkers		
CRP	0.234*	0.144
MMP-9	−0.002	0.168
E-Selectin	0.278**	0.089
sVCAM-1	0.155	0.089
sICAM-1	0.062	−0.082
Fibrinogen	0.251*	−0.027
D-dimer	0.115	0.067
PAI-1	0.307**	0.269*
MCP-1	0.260*	0.092
TSP-1	−0.100	0.157
MPO	0.237*	0.075
TGF-β	−0.003	0.077
SAA	0.275**	0.195
SAP	0.169	0.131
IL-1β	0.250*	0.198
IL-6	0.322**	0.146
IL-8	0.200	0.160
IL-10	0.397**	0.168
TNF-α	0.205	0.123

*p-value < 0.05.

**p-value < 0.01.

### Characterization of circulating LDGs in PLWH

Studies suggest that LDGs are a primary source of NETs due to an enhanced spontaneity for NET formation^39,44^. A recent study reported that LDGs from treatment-naïve PLWH displayed mature phenotypes and were also degranulated with higher CD63 and lower arginase 1 expression, suggesting an activated LDGs phenotype^38^. However, LDGs in ART-controlled PLWH are less well characterized. Utilizing an available subset of participants’ PBMC (PLWH; n = 37 and HIV−; n = 48), we assessed circulating LDG levels and their phenotype by flow cytometry analysis (Suppl. Fig. 1).

**Figure 2:**
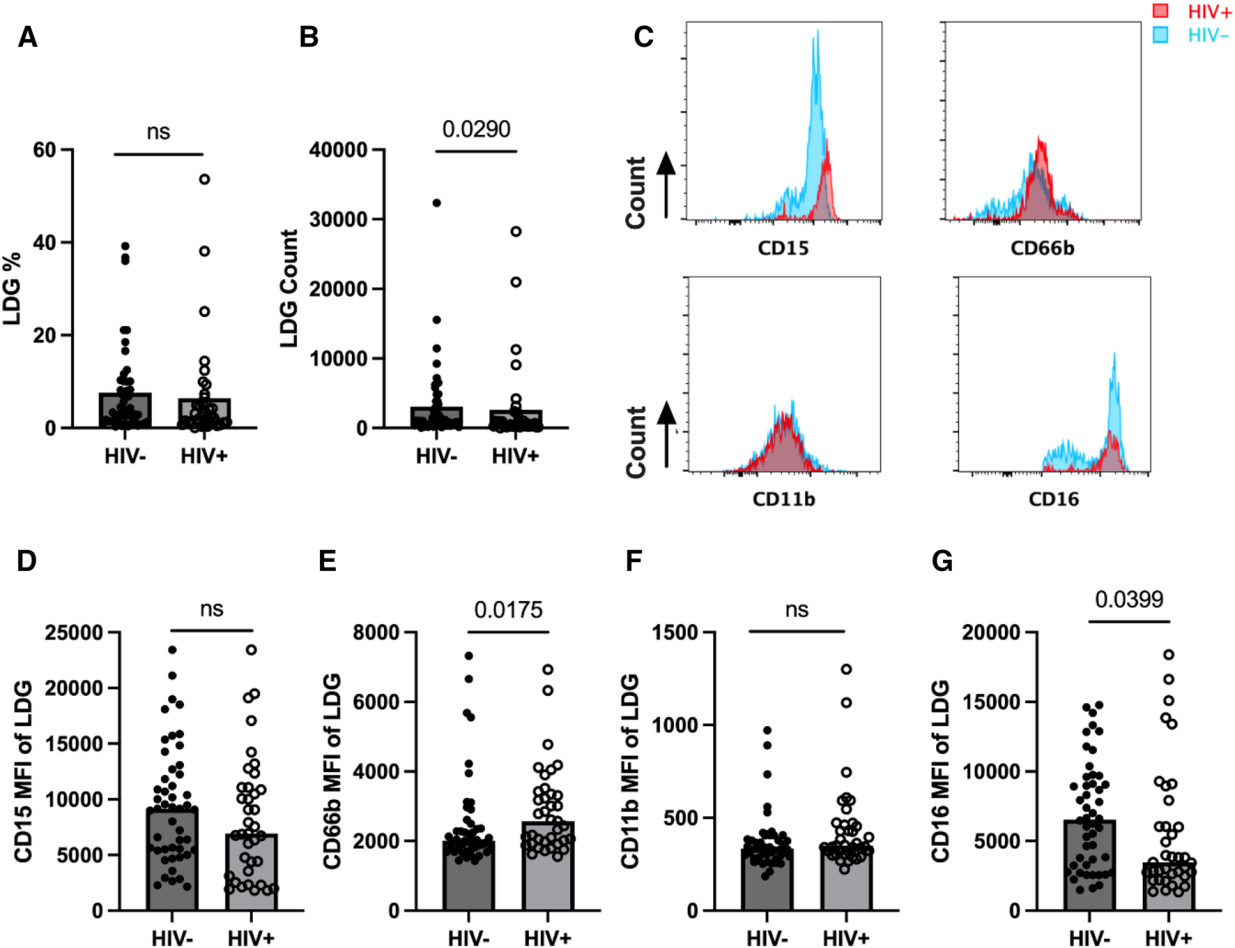
Characterization of LDG population in HIV+ (n = 37) and HIV− (n = 48) individuals (**A,B**). Percentage (**A**) and numbers (**B**) of LDGs in HIV− and HIV+. (**C–G**) Comparison of expression of neutrophil markers on LDGs between groups. Representative histograms of expression of CD15, CD66b, CD11b, and CD16 in LDGs in HIV− (blue) and HIV+ (red) groups (**C**). CD15 MFI (**D**), CD66b MFI (**E**), CD11b MFI (**F**), and CD16 MFI (**G**) of in HIV− and HIV+ individuals. Mann Whitney-U test; ns = p > 0.05.

The percentages of LDGs were comparable between groups; however, LDG counts were lower in PLWH than in HIV− individuals (Fig. 2A, B). LDG parameters were not associated with disease severity, such as CD4 count, CD4/CD8 ratio, or viral load (data not shown). Next, we analyzed the expression levels of neutrophil markers to determine if PLWH display altered phenotypes of LDGs compared with those of HIV− individuals. There was no significant difference in the median fluorescence intensity (MFI) of CD11b and CD15 observed between the groups, although a decreasing trend in CD15 MFI on LDGs was detected in PLWH (Fig. 2C, D, F). However, LDGs from PLWH had significantly decreased CD16 expression, but increased expression of CD66b, compared to HIV− individuals (p = 0.0399 and p = 0.0175, respectively) (Fig. 2E, G). These results suggest that ART-controlled PLWH display altered LDG phenotypes.

### Decreased mature CD10^+^ LDGs in PLWH

**Figure 3:**
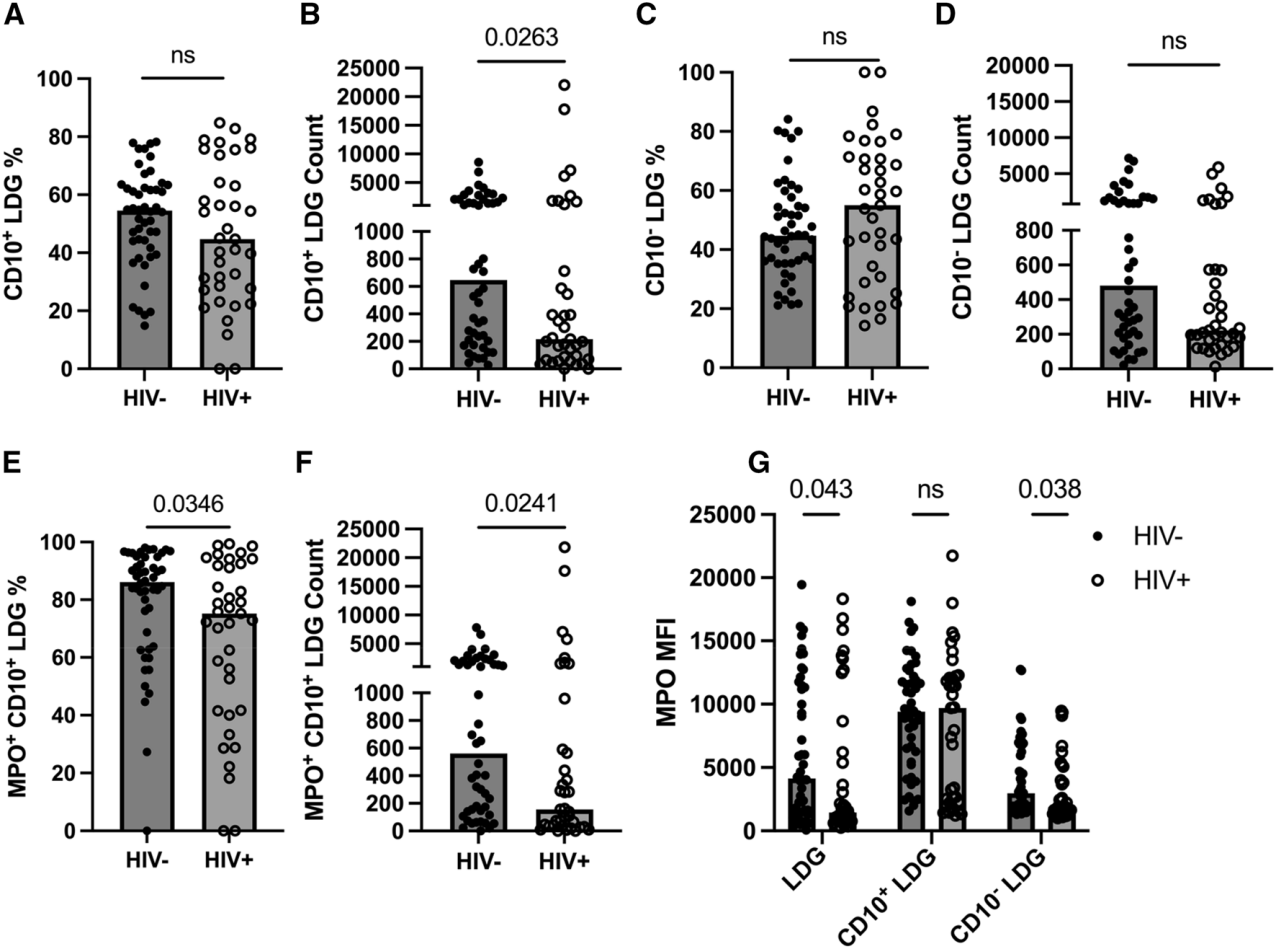
Decreased mature CD10^+^ LDGs were observed in PLWH. (**A–D**) The percentages and counts of CD10^+^ and CD10^−^ LDGs in HIV+ (n = 37) and HIV− (n = 48). CD10^+^ LDG percentage (**A**), CD10^+^ LDG count (**B**), CD10^−^ LDG percentage (**C**), CD10^−^ LDG count (**D**) in HIV− and HIV+ individuals. MPO expression in LDGs. MPO^+^ CD10^+^ LDG percentage (**E**), MPO^+^ CD10^+^ LDG count (**F**), and MPO MFI of LDG, CD10^+^ LDG, and CD10^−^ LDG in HIV− and HIV+ individuals (**G**). Mann Whitney-U test; ns = p > 0.05.

In PLWH, the observed decrease in CD15 and CD16 expression in LDGs compared to HIV− individuals was regarded as a more immature phenotype. This observation suggests that the maturation status of LDGs between PLWH and HIV− individuals differs, prompting us to assess the CD10 expression of LDGs. We observed decreased CD10^+^ LDG counts in PLWH compared to HIV− individuals. A decreasing trend in the percentage of CD10^+^ LDGs was shown in HIV+ group, albeit not statistically significant (Fig. 3A, B). However, the percentages and counts of CD10^-^ were not statistically different between groups (Fig. 3C, D).

Elevated immature neutrophils drive the pro-inflammatory response in various pathologic conditions, such as cancer, infection, and inflammatory diseases^29,45–47^. Thus, we further analyzed MPO^+^ cells among CD10^+^ LDG subsets to determine whether the activated phenotype in mature LDGs is altered in PLWH. Our data demonstrated that the percentages and counts of MPO^+^CD10^+^ LDGs were lower in PLWH than those in HIV− individuals (Fig. 3E, F). Pairwise comparison of MPO MFI in LDGs and LDG subsets (CD10^+^ LDGs and CD10^−^ LDGs) showed decreased MPO MFI in total LDGs and CD10^−^ LDGs from PLWH, compared to those from HIV− individuals (Fig. 3G**)**. Taken together, our data indicate that PLWH display decreased numbers of mature CD10^+^ LDGs. Interestingly, decreased activation status, measured via MPO MFI, was only present within immature LDGs in PLWH compared to HIV− individuals.

### Decreased CD16^Hi^ LDG subset in PLWH

**Figure 4:**
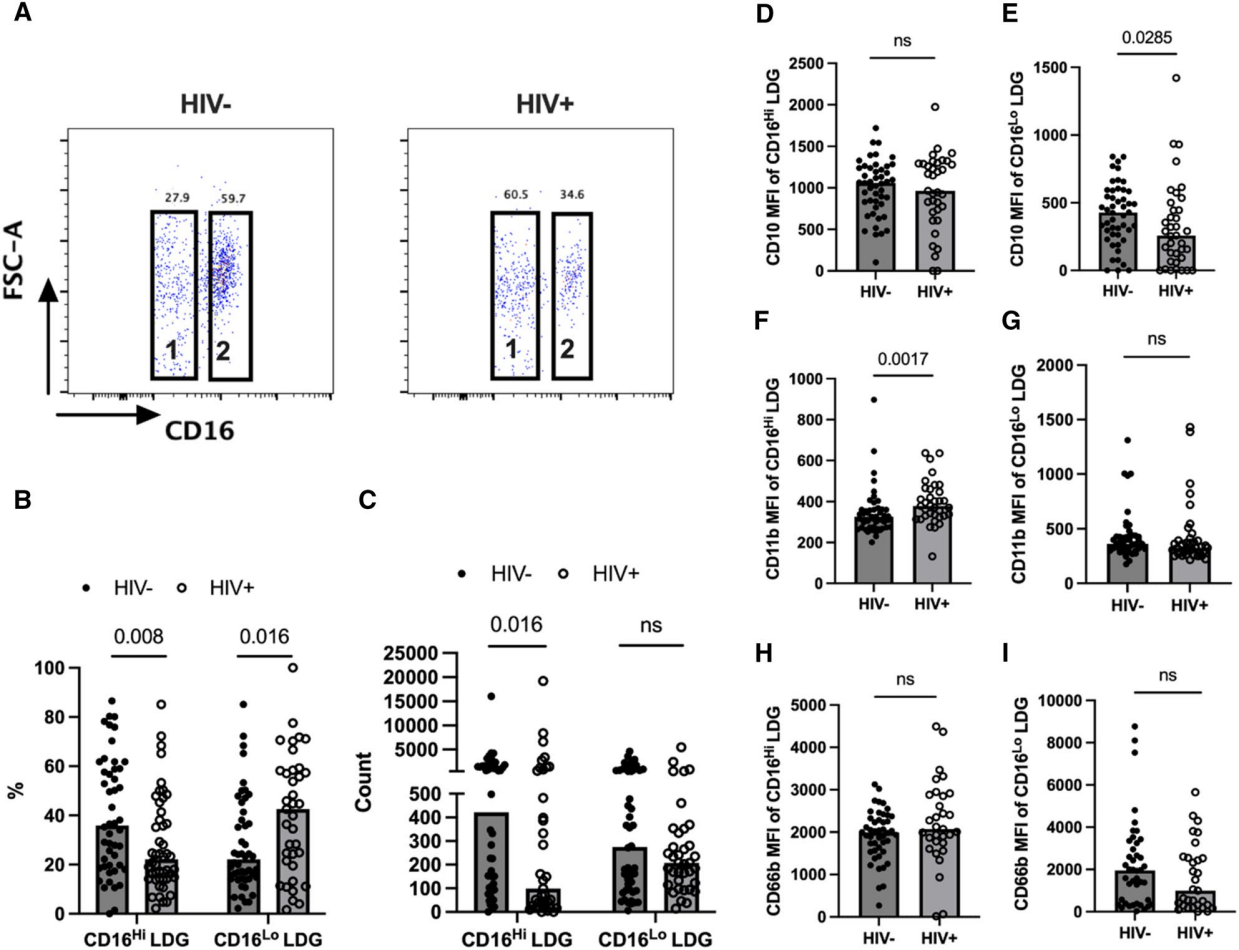
Comparison of CD16^Hi^ and CD16^Lo^ LDG subsets between groups. (**A**) Representative dot plot demonstrating CD16^Hi^ (2) and CD16^Lo^ (1) LDG populations in HIV+ (n = 37) and HIV− (n = 48) groups. (**B**) CD16^Hi^ and CD16^Lo ^LDG percentages in HIV− and HIV+ groups. (**C**) CD16^Hi^ and CD16^Lo^ LDG counts in HIV− and HIV+ groups. (**D–I**) CD10 MFI of CD16^Hi^ (**D**) and CD16^Lo^ LDG (**E**), CD11b MFI of CD16^Hi^ (**F**) and CD16 ^Lo^ LDG (**G**), CD66b MFI of CD16^Hi^ (**H**) and CD16^Lo^ LDG (**I**). Mann Whitney-U test; ns = p > 0.05.

Studies have revealed that neutrophils are heterogeneous and neutrophil subsets are distinguishable based on the expression levels of neutrophil markers, with subsets exhibiting distinct phenotypic and functional properties^48–50^. To gain insight into LDG heterogeneity in PLWH on stable ART, the distribution of CD16 expression on LDGs was analyzed and LDGs were divided into two subsets, CD16^Lo^ and CD16^Hi^ LDGs based on CD16 expression (Fig. 4A). In a similar situation as the decreased LDG CD16 MFI in PLWH (Fig. 2C, G), we observed decreased percentages and counts of CD16^Hi^ LDGs, but increased percentages of CD16^Lo^ LDGs (Fig. 4A, B, C). However, CD16^Lo^ LDG counts were comparable between groups (Fig. 4C). CD16^Hi^ mature neutrophils from HIV- individuals expressed higher CD11b than those from PLWH (Fig. 4F), but CD10 and CD66b expressions were not different between groups (Fig. 4D, H). In PLWH, the CD16^Lo^ LDG subsets resembled granulocyte precursors with lower CD10 expression (Fig. 4E) and tended to towards reduced CD66b expression (Fig. 4I). However, CD11b expression was comparable between groups (Fig. 4G). Our data demonstrate that mature neutrophils are the majority of cells found in HIV− individuals, while ART-controlled PLWH exhibit significantly decreased mature neutrophils in circulation.

### Characterization of NET forming LDGs in PLWH

**Figure 5:**
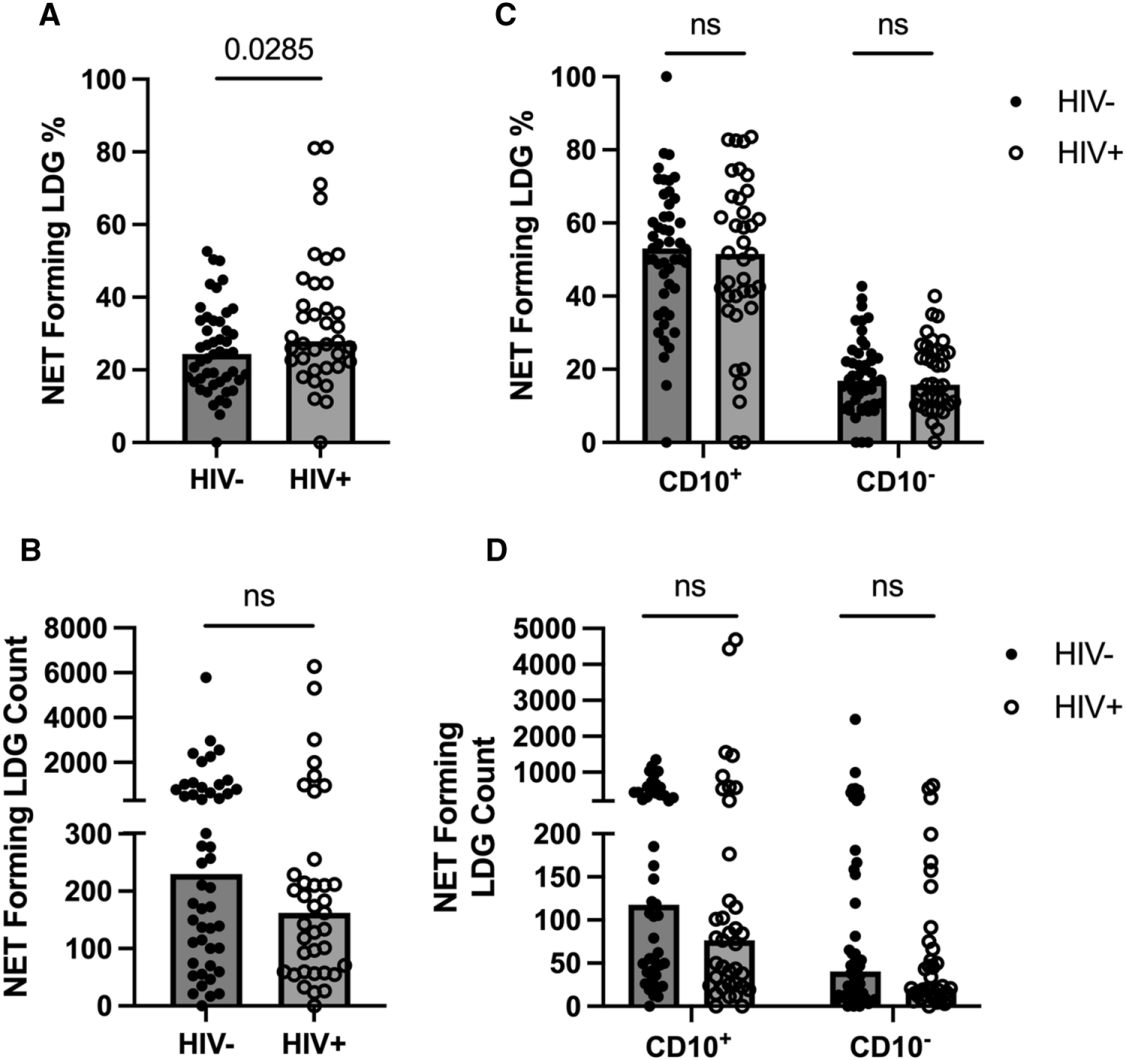
NET-forming LDGs in HIV+ (n = 37) and HIV− (n = 48) individuals. (**A,B**) NET forming LDG percentage (**A**), NET-forming LDG count (**B**) in HIV− and HIV+ individuals. (**C,D**) CD10^+^ and CD10^−^ NET-forming LDG percentage (**C**), CD10^+^ and CD10^−^ NET forming LDG count (**D**) in HIV− and HIV+ individuals. Mann Whitney-U test; ns = p > 0.05.

We found that CitH3 levels were significantly increased in the plasma of PLWH concurrently with a decreased mature LDG count. These findings prompted us to examine NET-forming LDG in PLWH and HIV- groups and assess if LDGs from PLWH are more prone to NET formation. First, we measured the MFI of side scatter (SSC) signal on LDGs to determine cell size and granularity differences between PLWH and HIV− individuals. SSC-A MFI were comparable between groups (Suppl. Fig. 2). To identify NET-forming LDGs, CitH3 and MPO double-positive cells were analyzed by flow cytometry (Suppl. Fig. 1). We found that the percentage of NET-forming LDGs was increased in PLWH compared with HIV− individuals although NET forming LDG counts were comparable between groups. (Fig. 5A, B). Although LDGs have been reported to be a source of NET production, whether immature and/or mature neutrophil populations are more or less responsible for such NET capacity has never been investigated. To define LDG subsets as responsible for NET formation, we divided NET-forming LDGs into CD10^−^ vs CD10^+^ between groups. Results showed that the percentage and count of NET forming cells were not different regardless of CD10 expression (Fig. 5C, D).

### Inflammatory cytokines and neutrophil activation markers correlate with LDGs in PLWH

In order to determine whether LDGs and their NET formation correlate with cytokine expression in PLWH, we performed spearman rank correlation to assess associations between cytokines and LDG subset (LDG, CD10^+^ LDG, and NET-forming LDGs) counts and percentages. In Table 3, LDG % was positively correlated with TSP-1, MMP-9, MPO, TGF-β, and IL-1β. LDG counts positively correlated with TSP-1, MMP-9, and TGF-β. CD10^+^ LDG % and the counts were positively associated with TSP-1 and TGF-β levels, but CD10^+^ LDG % and the counts were negatively correlated with IL-6 and IL-8, respectively. NET-forming LDG counts were positively associated with TSP-1 and MMP-9, but negatively associated with IL-10 (Table 3).

**Table 3:** Spearman’s correlations between soluble markers and LDG parameters in PLWH.

Soluble markers	LDG parameters	R value
TSP-1	LDG %	0.458**
	LDG count	0.386*
	CD10^+^ LDG %	0.418*
	CD10^+^ LDG count	0.490**
	NET-forming LDG count	0.427**
MMP-9	LDG %	0.568**
	LDG count	0.459**
	CD10^+^ LDG count	0.505**
	NET-forming LDG count	0.508**
IL-6	CD10^+^ LDG %	−0.413*
	CD10^+^ LDG count	−0.344*
IL-8	CD10^+^ LDG %	−0.351*
IL-10	NET-forming LDG %	−0.444**
	NET-forming LDG count	−0.333*
MPO	LDG %	0.415*
TGF-β	LDG%	0.380*
	LDG count	0.340*
	CD10^+^ LDG count	0.398*
IL-1β	LDG %	0.371*

*p < 0.05.

**p < 0.01.

## Discussion

Elucidating biological mechanisms underlying NACM development in PLWH in the era of successful and sustained ART is required for mitigating comorbidity risk and progression. Neutrophils and their ability to release NETs have been well-documented as one of the defense mechanisms against pathogens^20–22,49^. Far beyond their antimicrobial role, a growing body of evidence over the last decade has demonstrated that uncontrolled and/or excessive NET formation is associated with deleterious effects on disease development or progression^27,28,39,48^. Sivanandham et.al., has documented that increased NETs are observed during SIV infection, even in non-human primates administered an ART regimen^51^. In addition, altered platelet aggregation spurred on by NET formation and tissue factor expression on NET structures supports evidence that NETs impact coagulation in SIV, and therefore HIV infection^51^. The detrimental role of NETs in ART-controlled PLWH still largely remains unknown. In addition, understanding the inflammatory response underlying NETs in PLWH remains incomplete. Here, we analyzed circulating levels of NET markers in PLWH on ART and compared the plasma NET levels with a cohort of HIV seronegative individuals. To the best of our knowledge, there is currently no report assessing NETs in PLWH with sustained ART treatment. We demonstrate for the first time that plasma CitH3 levels were significantly elevated in PLWH. In addition, plasma CitH3 levels were positively associated with inflammatory markers linked to CVD, a primary NACM in PLWH. This observation can be further aimed at characterizing neutrophils to understand differential phenotypic and functional properties between PLWH and HIV− individuals, as well as their contributions to NET formation.

As neutrophils are sources of NET production, NET levels were correspondingly associated with the elevation of WBC and neutrophil counts. Data regarding blood counts were comparable between groups, suggesting that the observed increase in CitH3 levels resulted from neutrophil activation, not an increased cell population at baseline. Intriguingly, there was no significant difference in cfDNA levels between groups, although a positive association between cfDNA and CitH3 was demonstrated in PLWH. Furthermore, the association analysis revealed that cfDNA, but not CitH3, was positively correlated with leukocyte and absolute neutrophil counts. These results support the possibility that cfDNA production may not be exclusive to neutrophil-related NETs and may be impacted by other mechanisms of cfDNA release.

Several studies have demonstrated that ART can help to reduce inflammation by preventing viral replication, but inflammatory biomarker profiles do not always return to pre-HIV infection levels^52–54^. Our data demonstrates that CitH3 levels were positively correlated with IL-1β, IL-6, IL-10, and MPO in PLWH. This is consistent with reports that demonstrate IL-10 expression correlates with HIV viremia^55,56^, supporting a role for IL-10 in favoring the persistence of inflammation in PLWH on ART. Our data suggests that chronic inflammation in PLWH can increase neutrophil predisposition to form NETs. NETs may initiate a positive feedback loop by secreting neutrophil granules and promoting cytokine production and release from other cell types. Interestingly, we found that plasma levels of IL-10 were negatively correlated with the percentages and counts of NET-forming LDGs in PLWH. Our finding is corroborated by a recent publication by Saitoh et. al., who utilized an in vitro analysis of NETs with anti-IL-10-neutralizing antibody and co-culture of neutrophils and dendritic cells demonstrated that CD209-dependent production of IL-10 by dendritic cells inhibited HIV-1-induced NET formation^22^. A recent study showed that CitH3 expression was detected in activated neutrophils without NET formation^57^, suggesting that citrullination of histones in neutrophils is unlikely to be solely responsible for NET formation. Thus, we assume that sources of elevated CitH3 levels in PLWH result from both NETosis and NET-independent neutrophil activation. Given the relationship between inflammatory cytokines and CitH3 in our study, it is necessary to identify inflammatory stimuli associated with NET formation in HIV infection. In line with findings of NET-induced thrombotic effects in SIV infection, our data demonstrates that plasma levels of CitH3 were positively associated with coagulation markers, fibrinogen, SAA, and PAI-1. These data suggest that neutrophil activation promotes thrombogenesis in HIV. Further studies are required to understand the mechanism underlying thrombosis regulated by NETs in HIV.

At homeostasis, CD16 and CD62L expression in circulating neutrophils are maintained within narrow ranges, but these expressions change during inflammatory conditions. The expression of CD16 has been found to decrease in LDGs in individuals with RA and antineutrophil cytoplasmic antibody (ANCA) positive vasculitis^44^. Recently, LDGs expressing intermediate levels of CD16 were demonstrated to become enriched in severe COVID-19 patients^58^. A study by Cloke et al*.* found that the morphology of LDG from treatment naïve HIV-infected individuals was similar to normal-density granulocytes (NDG), and that LDG became increasingly activated with a mature phenotype^42^. Similar to other reports^36^, we found that LDGs from ART-controlled PLWH were a heterogenous population of mature and immature neutrophils, but that the relative proportion of immature (CD10^-^) LDGs trended higher in PLWH. LDGs from PLWH displayed immature and activated phenotypes^42^ as the cells expressed decreased CD10 and CD16, but increased CD66b. Furthermore, a marked decreased of MPO expression on LDGs and CD10^-^ LDGs in PLWH suggests that degranulation and/or phagocytic activities of LDGs are altered in immature LDG populations. It has been demonstrated that the majority, but not all, immature neutrophils from patients with sepsis show an increase in spontaneous NET release compared to NDG from healthy individuals. Interestingly, these same cells do not release NET production upon PMA stimulation^59^. We found that LDGs from PLWH had increased NET forming efficacy regardless of maturation status. These results suggest that immature LDGs in PLWH show a propensity for NET formation rather than degranulation and/or phagocytosis. Further ex vivo NET assays using neutrophils isolated from PLWH will require to determine if elevated NETs in PLWH are due to cells’ increased susceptibility to form NETs spontaneously. Elucidating if ART-controlled individuals present with a pro-inflammatory-skewed milieu favoring NET formation and understanding neutrophil heterogeneity in those population are important avenues for future studies.

In summary, we found that ART-controlled PLWH had increased CitH3 expression and immature LDGs with enhanced NET forming efficacy and reduced MPO expression. Our study showed that altered systemic cytokine environment in PLWH on ART could affect neutrophil activation and NET formation. Additionally, increased NET levels were positively associated with markers for proinflammation and coagulation, suggesting NETs can be detrimental in chronic inflammation and thrombogenesis in PLWH.

## Methods

### Study participants

This cross-sectional study analyses used data and banked PBMCs from the entry time point of longitudinal Hawaii Aging with HIV-Cardiovascular (HAHC) study. The study design and enrollment details have been published previously^60^. In brief, inclusion criteria for the parent study required participants to have completed > 3 months of potent ART prior to enrollment. A cohort of HIV− individuals was recruited as a comparator group. The study was approved by the University of Hawaii Committee on Human Subjects (CHS #16476, and #17857). Written informed consent was obtained from all participants. All experiments were performed in accordance with the relevant guidelines and regulations.

### Clinical parameters

Clinical parameters, including height, weight, and BMI were calculated as (weight in kilograms)/(height in meters)^2^. Blood pressure (systolic and diastolic blood pressure) were obtained as triplicate average. T cell subsets (CD4^+^/CD8^+^) and plasma HIV RNA assessment were performed. Undetectable plasma HIV RNA was defined as HIV RNA ≤ 50 copies/ml. Chemistry and metabolic labs [glucose, insulin, total cholesterol high-density cholesterol (HDL-C) and low-density cholesterol (LDL-C), and triglycerides] were obtained at entry in a fasted state. Subjects were assessed for health behaviors (smoking and alcohol intake), medication use, and health outcomes.

### Detection of NET and soluble biomarkers in plasma

Circulating cfDNA in the plasma was quantified using the Quant-iT™ PicoGreen™ dsDNA Assay-Kit (ThermoScientific, P11496) according to the manufacturer's instructions. The fluorescence was read in a VICTOR3 plate reader (Perkin Elmer) by fluorescence 485 nm/535 nm, 1.0 s protocol. The concentration of the circulating cfDNA was calculated based on the standard provided by the kit. CitH3 was quantified in plasma using the enzyme-linked immunosorbent assay according to the manufacturer’s instructions (Cayman, 501620). We used previously generated plasma cytokine data to assess associations of circulating NET markers and LDG parameters with soluble biomarkers in PLWH. List of cytokines in Table 2 was measured via a MILLIPLEX^®^ Human Cardiovascular Disease panels (EMD Millipore) according to the manufacturers protocol.

### Flow cytometric analysis

Cryopreserved PBMCs were rapidly thawed at 37 °C and immediately transferred to pre-warmed complete media. After centrifugation, 1–2 × 10^6^ PBMC were incubated with Fixable Viability Dye eFluor 506 (Ebiosciences, 1:1000) at 4 °C for 30 min protected from light. Cells were washed twice in flow buffer (HBSS supplemented with 1% BSA) and the pellet was resuspended in 50 μL of Human TruStain FcX (BioLegend, San Diego, CA, 1:200) in flow buffer at RT for 15 min, protected from light. Subsequently, cells were stained with the titrated fluorophore-conjugated primary antibody cocktail (Suppl. Table 1) at RT for 30 min, protected from light, and then washed twice with ice-cold flow buffer. For the intracellular staining, cells were resuspended in 250 µL of BD Cytofix/Cytoperm (BD Technologies, East Rutherford, NJ) for 30 min at 4 °C and then incubated with the titrated primary anti-MPO-PE and primary CitH3 antibody (Abcam, Waltham, MA) for 30 min at 4 °C, protected from light. Allophycocyanin (APC)-conjugated anti-goat secondary antibody (Invitrogen, Waltham, MA) at 1:500 dilution was added for labeling the anti-CitH3 primary antibody. Samples were then washed twice with ice-cold flow buffer and resuspended to 800 μL of flow buffer for acquisition. Samples were acquired on an Attune NxT Flow Cytometer (Thermofisher, Waltham, MA) with approximately 1.0 × 10^6^ events collected per sample. Volumetric calculations directly determined cell counts by the Attune Nxt (Thermofisher, Waltham, MA) machine. Data analysis was performed using FlowJo Version 10.8.1 (Treestar, Ashland, OR) software. LDG were defined as CD45^+^/CD11b^+^/CD14^-^/CD16^+^/CD15^+^ cells. NET forming LDG were defined as CD45^+^/CD11b^+^/CD14^-^/CD16^+^/CD15^+^/MPO^+^/CitH3^+^ cells.

### Statistical analyses

Categorical variables between PLWH and HIV-seronegative controls were compared using chi-square test of proportions or Fisher’s exact test, as appropriate. Continuous variables, including levels of CitH3 and cfDNA were compared using the non-parametric Mann Whitney-U test. Spearman’s rho was used to assess correlations between plasma soluble biomarkers and markers of NET formation. P-value < 0.05 was considered statistically significant for all tests. Statistical analysis was performed SSPS Version 29.0 and figures created with corresponding p-values using Prism 9 (Graph Pad, San Diego, CA).

### Supplementary Information

Supplementary Figures.

Supplementary Table 1.

## Data Availability

The datasets generated and deidentified participant data will be made available upon reasonable request.
