# Supplementary material for: Low-density granulocytes display immature cells with enhanced NET formation in people living with HIV
Source: Sci Rep. 2023 Aug 16;13:13282. doi: 10.1038/s41598-023-40475-0 (PMC10432506; doi:10.1038/s41598-023-40475-0)
Supplement: Supplementary file 1 — Supplementary Figures. [file 41598_2023_40475_MOESM1_ESM.docx]

**Supplementary Figure 1**


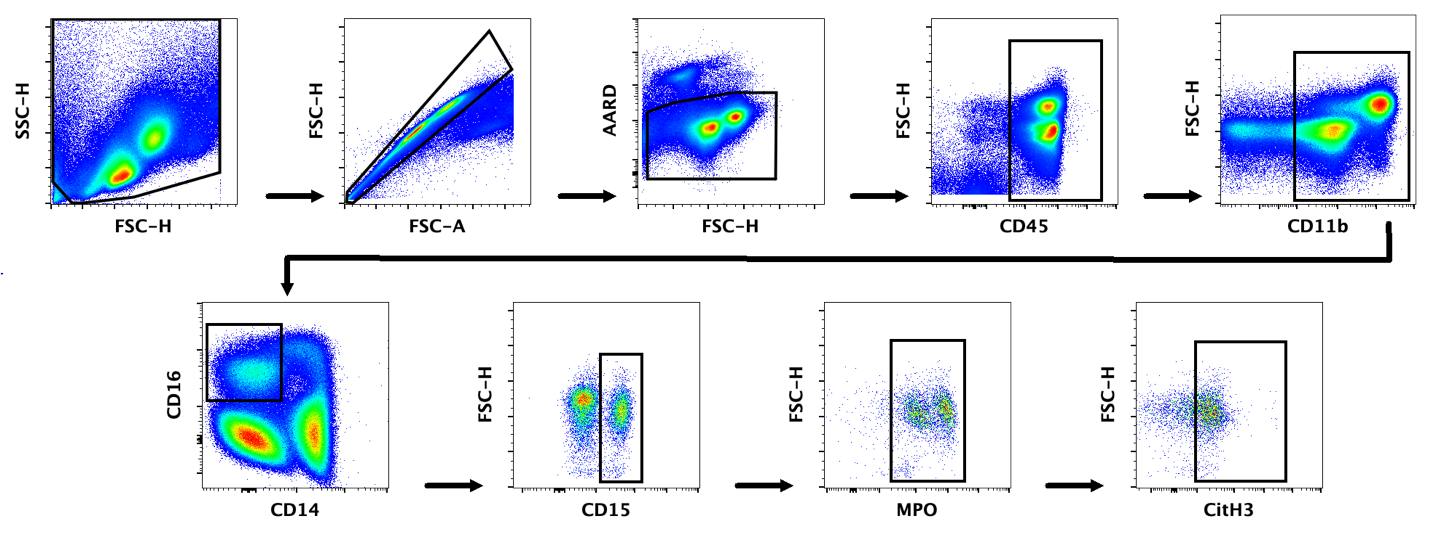


Supplementary Figure 1: Representative gating strategy for identification of NET forming LDG populations from whole PBMC specimens.

**Supplementary Figure 2**


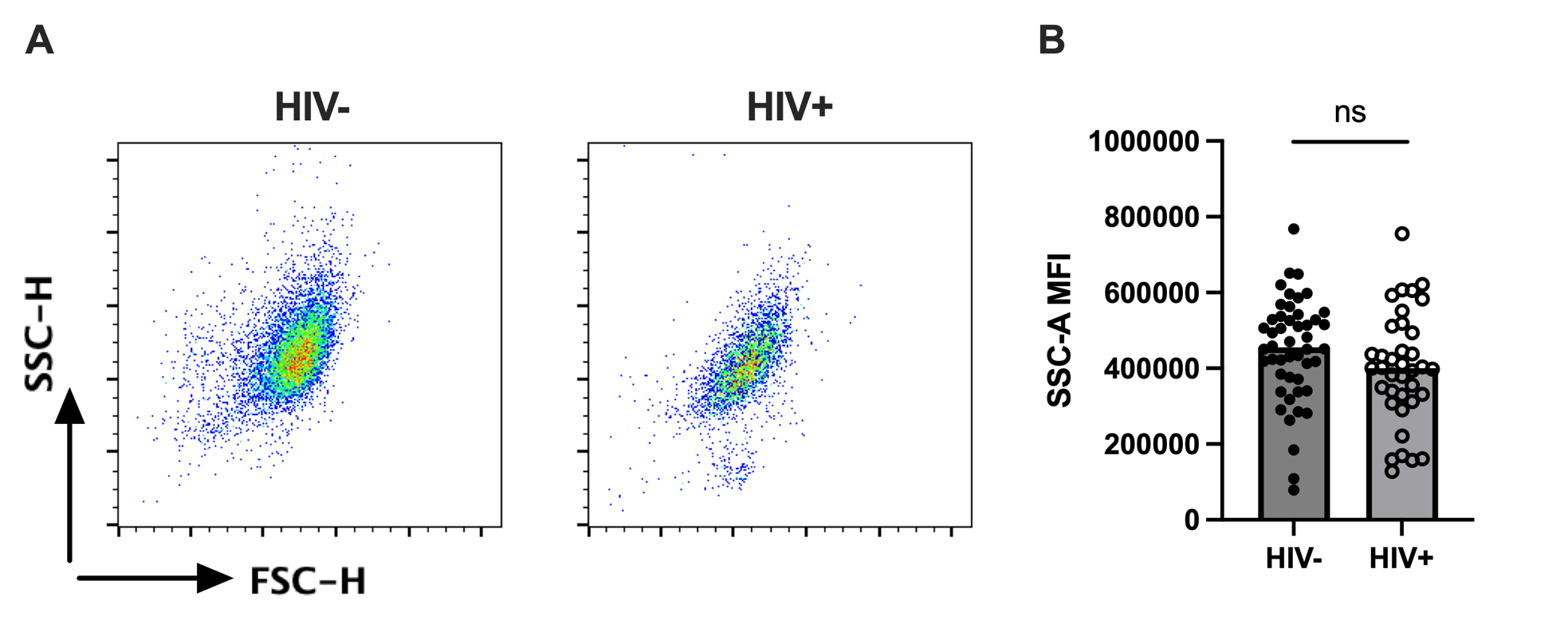


Supplementary Figure 2: **A**. Representative dot plot demonstrating the size and granularity characteristics of LDGs in HIV- and HIV+ groups **B.** Graphical representation of granularity (visualized via side scatter area) in HIV- and HIV+ groups. Mann Whitney-U test; ns = p > 0.05
