# Supplementary material for: Low-density granulocytes display immature cells with enhanced NET formation in people living with HIV
Source: Sci Rep. 2023 Aug 16;13:13282. doi: 10.1038/s41598-023-40475-0 (PMC10432506; doi:10.1038/s41598-023-40475-0)
Supplement: Supplementary file 2 — Supplementary Table 1. [file 41598_2023_40475_MOESM2_ESM.docx]

**Supplementary Table 1.** Antibody-fluorophore conjugations and company used for flow cytometry

| **Antibody Target** | **Conjugate Fluorophore** | **Company** |
| --- | --- | --- |
| CD45 | BV711 | BD Biosciences (East Rutherford, NJ) |
| CD11b | PE-Cy-7 | BioLegend (San Diego, CA) |
| CD14 | BV605 | BioLegend (San Diego, CA) |
| CD16 | BV650 | BioLegend (San Diego, CA) |
| CD15 | FITC | Millipore Sigma (St. Louis, MO) |
| CD10 | PerCP5.5 | R&D Systems (Minneapolis, MN) |
| CD41 | PE-Dazzle | BioLegend (San Diego, CA) |
| CD62p | AF700 | BioLegend (San Diego, CA) |
| CD66b | BV421 | BioLegend (San Diego, CA) |
| MPO | PE | BD Biosciences (East Rutherford, NJ) |
| citH3 | APC | Abcam & Invitrogen (Waltham, MA), respectively |
| Viability | eFluor506 | Invitrogen (Waltham, MA) |
